# Mammoth Giant Cell Tumor of the First Metacarpal: A Case Report and Management Trends

**DOI:** 10.7759/cureus.16150

**Published:** 2021-07-03

**Authors:** Alok C Agrawal, Shilp Verma, Bikram Kar, Harshal Sakale, Ranjeet Choudhary

**Affiliations:** 1 Orthopedics, All India Institute of Medical Sciences, Raipur, IND

**Keywords:** giant cell tumor, dual joint fusion, fibula graft, first metacarpal, complete excision and reconstruction

## Abstract

Giant cell tumor (GCT) is classically described as a locally aggressive, epiphyseo-metaphyseal osteolytic tumor occurring in young adults. They are mostly seen in long bones while some are also found in the iliac bone and spine and a very small proportion occurs in hand bones. Due to the rarity of GCT in metacarpal, there is a paucity of treatment options available. In an extensive literature search on PubMed, Embase, Medline, and Ovid from 2004 till date, very few cases were reported.

The various treatment options available are intralesional curettage with or without adjuvant therapy, wide resection, free osteoarticular metatarsal transfer, and, occasionally, ray amputation may also be done. After simple curettage, a reasonably high recurrence rate also imposes comprehensive en-bloc excision, but still, there are many case reports of recurrence.

Experience with a case of GCT of the whole first metacarpal extending from the carpometacarpal to the metacarpophalangeal joint is not thoroughly described in the literature. We hereby report a mammoth GCT of the first metacarpal treated by excision and reconstruction by free fibular graft and adjacent joint fusion with an excellent functional outcome at one-year follow-up.

## Introduction

Giant cell tumor (GCT) is described as a locally aggressive, osteolytic tumor occurring in young adults at the epiphyseal region. Around 80%-90% of giant cell tumors are seen in long bones, 4% in the iliac spine and iliac bone, and only 2% of giant cell tumors are described in hand. (the phalanges, rarely, are located within the thumb and metacarpals). Unni reports an incidence of 1.7% for giant cell tumors of metacarpals [1]. According to Averill et al., less than 1.5% of giant cell tumors are reported in metacarpal [2]. Due to the rarity of GCT in metacarpal, there is a paucity of treatment options available. In an extensive literature search from 2004 till date, only 22 cases were reported.

The various treatment options available are intralesional curettage with or without adjuvant therapy, wide resection of the tumor, followed by joint reconstruction by allograft or arthroplasty, joint arthrodesis augmented with bone graft, free osteoarticular metatarsal transfer, and, occasionally, ray amputation. Initially, after curettage followed by autologous bone graft, around 90% of recurrence was reported [3], leading to extensive en block excision of the tumor as the only option [4]. We hereby report our experience with a case of GCT of the whole first metacarpal extending from carpometacarpal to metacarpophalangeal joint and discussing all possible treatment options available for metacarpal GCT.

Most of the GCT of the first metacarpal is small and retractable. We are reporting a case of mammoth GCT of the first metacarpal, which was treated by excision and reconstruction by free fibular graft and adjacent joint fusion.

## Case presentation

A 29-year-old female came to the orthopedic outpatients' department (OPD) with a complaint of swelling of her left thumb with global restriction of thumb movement. The swelling was gradually progressive and associated with pain. There was no history of any constitutional symptoms or any injury. Initially, the patient had taken an indigenous system of treatment in the form of local ointment and heat application and developed a skin reaction; thereafter, the patient came to the orthopedics department. The patient had undergone a core needle biopsy with the diagnosis of GCT on histopathology. On physical examination, there was localized swelling over the right first metacarpal, of size around 10 x 7 x 4.5 cm, with variable consistency. The overlying skin was scarred with adhesion on the needle biopsy site, and the movements of the metacarpophalangeal (MCP) and trapeziometacarpal joints were painful and restricted.

**Figure 1:**
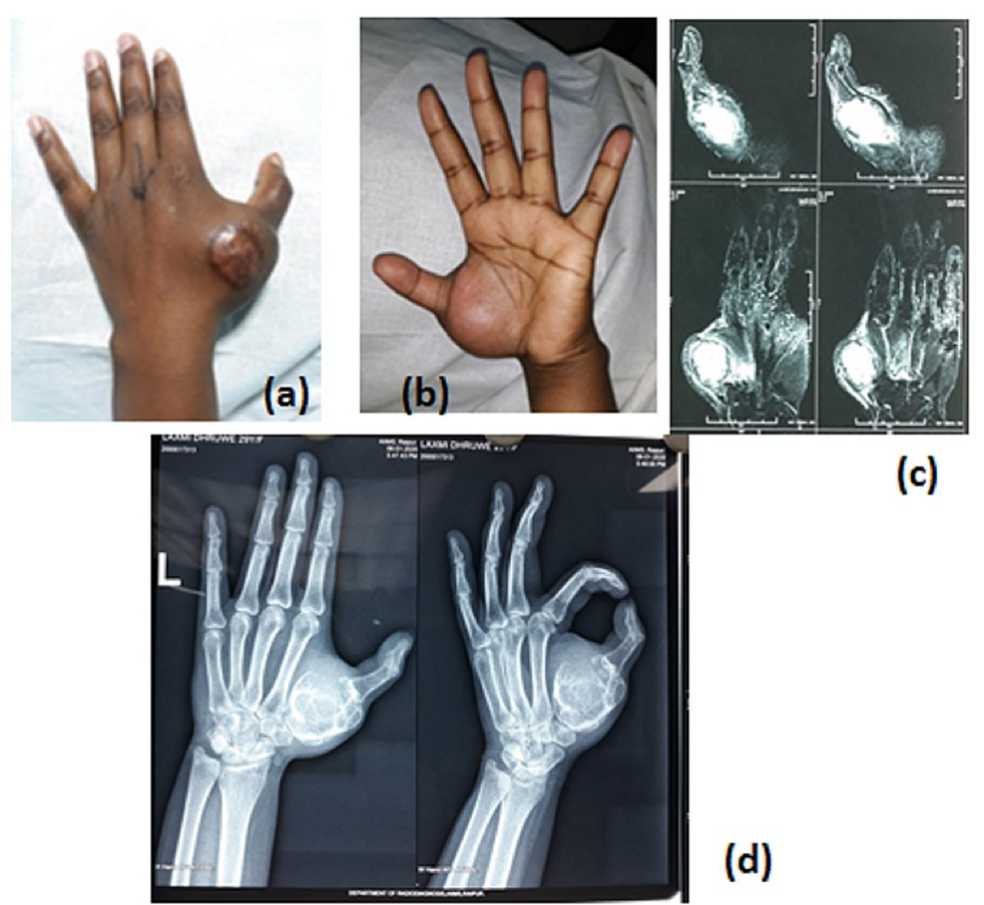
(a, b) Clinical picture of the left thumb, (c) MRI, and (d) radiograph showing expansile osteolytic lesion of the first metacarpal

Roentgenogram revealed an expansile osteolytic lesion of the first metacarpal in totality with a pathological fracture (Figure 1, panel d). A magnetic resonance scan (Figure 1, panel c) showed a 7.6 x 6.6 x 4.2 cm mass, which involved the whole first metacarpal in totality. The swelling was adherent to the skin and lesion on the biopsy site near the MCP joint. It was decided to excise the involved metacarpal in totality and reconstruct the thumb by a free fibula graft and adjacent joint fusion.

**Figure 2:**
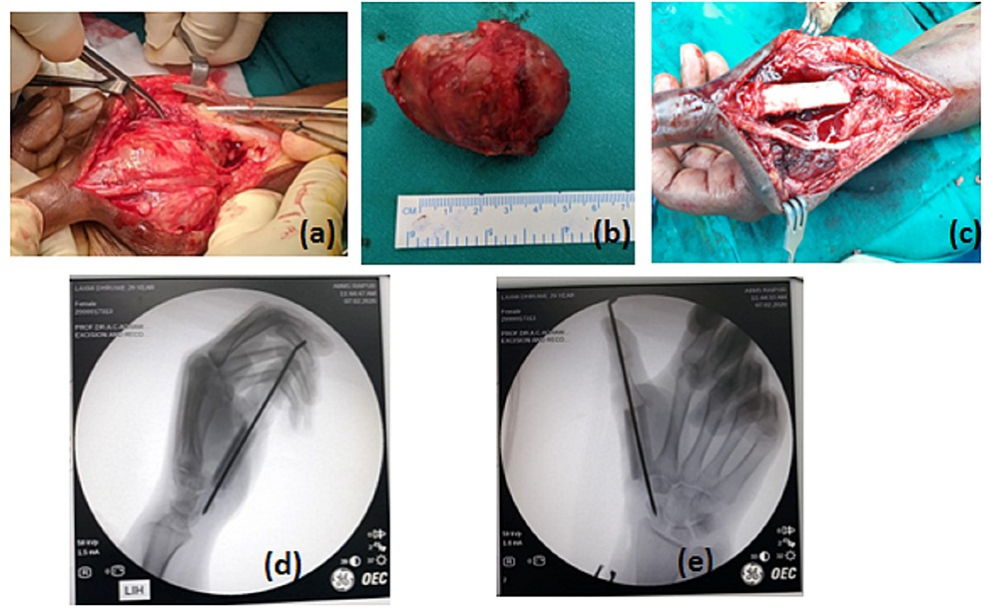
(a) Intraoperative tumor with extensive soft tissue involvement. (b) En-block resection of the GCT. (c-e) Clinical and IITV image with transfixed free fibula bone graft. IITV: image intensifying television

A dorsal longitudinal incision was given, extending from the first carpal metacarpal joint to the first proximal phalanx, crossing over the center of swelling. The skin and tendons over the swelling were retracted and extension tendons were cleared by a sharp knife and thermocautery. En-block resection of the tumor was done. The same length of the diaphyseal fibula was excised from the middle one-third of the left leg. To promote the union process, the articular surface of the trapezium was shoveled to expose good cancellous bone for fusion. A free fibular graft of size of the first metacarpal was fixed between proximal phalanx and trapezium in 40-degree abduction and flexion with the help of 2.6 mm transarticular Kirschner wire to achieve arthrodesis of the trapezium and proximal phalanx with fibula graft (Figure 2) in the functional position. The fibula graft was covered by the surrounding interosseous muscles and the incision site was closed in layers. A unilateral thumb spica slab was given postoperatively and was replaced by a spica cast after suture removal on the 14th day after surgery.

**Figure 3:**
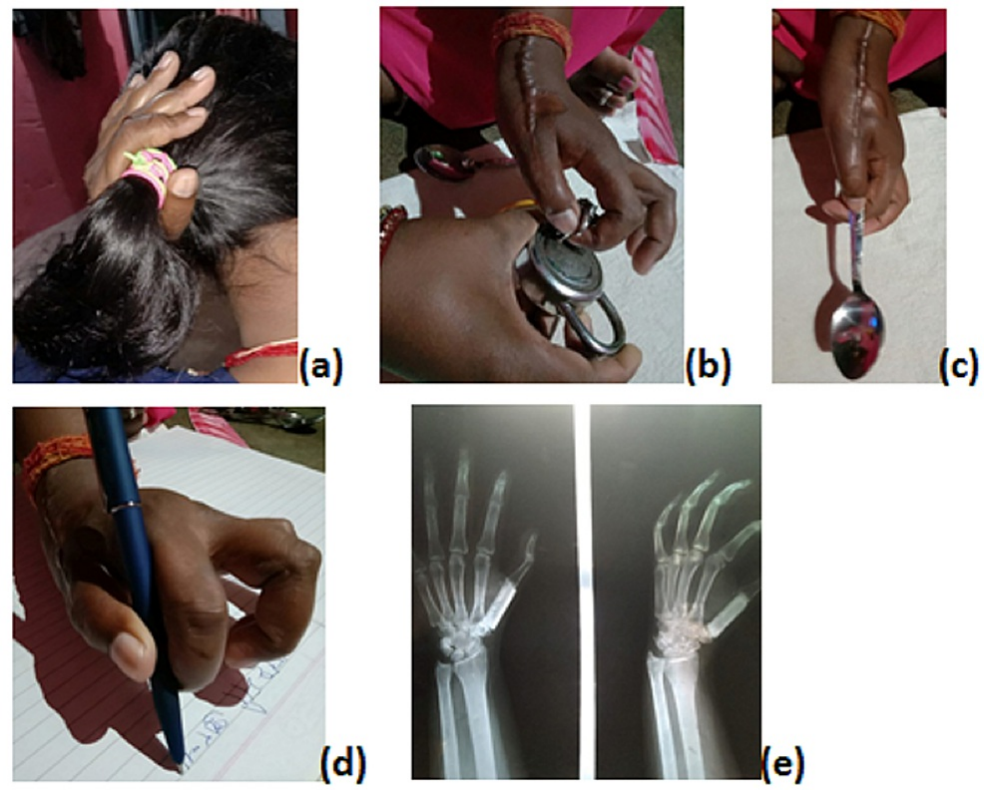
(a) One-year follow-up radiograph showing well-accepted fibula and adjacent joint fusion. (b) Functional thumb with span grip. (c,d) functional pinch grip, (e) functional ventral clenched grip.

Postoperatively, the excised specimen was confirmed as GCT on histopathological examination. The Kirschner wire, along with a thumb spica cast, was removed three months after the surgery. Thumb and wrist range of motion exercises were then started. Postoperative radiograph at 12 months showed a well-accepted fibula graft fused distally with first proximal phalanx and proximally with trapezium, with not a radiological sign of tumor recurrence and the patient has an optimum functional thumb (Figure 3).

## Discussion

Giant cell tumor accounts for about 4%-5% of all bone tumors and around 20% of benign bone malignancies. Most GCT are locally aggressive, but their course of progression is unpredictable, as many cases of distal metastasis are also reported in the literature [5]. GCT is mainly seen in long bone (around 75%-90% in the distal femur followed by 25% in the upper end of tibia, distal radius, and humerus [1] and only 1.7% to 4% in hand bones [1-3].

GCT of the hand are more locally aggressive, so in comparison to other long bone giant cell tumors, the signs and symptoms appear rapidly and expound even in the younger patients.

GCT usually present with clinical features of swelling and pain. It affects typically the epiphysis and epiphysis metaphysis region of the metacarpal. The differentials include aneurysmal bone cyst (ABC), non-ossifying fibroma, and osteosarcoma [6-7]. A management protocol of the giant cell tumor in metacarpal has not progressed very much in the past few decades, due to the less availability of cases in the literature. Versatile modalities available with us are intralesional curettage with or without adjuvant therapy, wide resection, joint reconstruction techniques, including total joint replacement, allograft reconstruction, joint arthrodesis augmented with bone graft, free osteoarticular metatarsal transfer, and, occasionally, ray amputation.

We reviewed 23 (27 patients) case reports of metacarpal GCT (including ours) that have been published in the English literature from 2004 till now. Eleven patients were male, and 16 were female, with 19 on the right hand and eight on the left. GCT in the first metacarpal was in eight patients, second metacarpal in five patients, third metacarpal in one patient, fourth metacarpal in eight patients, and five patients with fifth metacarpal involvement. Metacarpal GCT is most commonly noted in the second and third decades of life (Table 1).

**Table 1:** Reported cases of metacarpal GCT GCT: giant cell tumor

	Series	Number of case	Age	Sex	Side	Site	Treatment of the reported case
01	A Chatterjee, D. B. Dholakia, and S. V. Vaidya, 2004 [8]	01	18 year	Male	Right	1^st^ metacarpal	Excised en masse and replaced with a silastic spacer
02	Marco Manfrini, et al. 2004 [9]	01	32 year	Male	Right	4^th^ metacarpal	En mass resection with fibular autograft and silicone implant arthroplasty
03	Tatsuya Yoshida, et al. 2007 [10]	02	7 year	Female	Right	2^nd^ metacarpal	Curettage, followed by phenol and ethanol application and then bone grafting
			23 year	Male	Right	4^th^ metacarpal	
04	P. P. Kotwal, C. Nagaraj, and V. Gupta, 2008 [11]	02	30 year	Female	Right	2^nd^ metacarpal	Marginal excision of the tumor followed by reconstruction with a reversed vascularised toe joint transfer
			32 year	Female	Right	2^nd^ metacarpal	
05	Mounir Arroud, et al. 2010 [12]	01	8 year	Male	Right	4^th^ metacarpal	Complete resection of the fourth metacarpal bone and reconstructed with a free fibular graft
06	Mohammad Shahid, et al. 2011 [13]	01	30 year	Male	Right	1^st^ metacarpal	En bloc resection with morselised iliac corticocancellous bone grafts
07	Patrick Jaminet, et al. 2010 [14]	01	28 year	Male	Right	2^nd^ metacarpal	Reconstruction with free vascularized scapular bone flap combined with nonvascularized free osteocartilaginous grafts from the second toe
08	Hunaina Al-Kindi, et al. 2011 [15]	01	34 year	Female	Right	1^st^ metacarpal	Curettage followed by local resection and bone grafting
09	Neil F. Jones, et al. 2012 [16]	01	66 year	Female	Right	4^th^ metacarpal	Metacarpal and MCP Joint reconstruction using a fibular osteocutaneous free flap and silicone arthroplasty
10	Jin Chang Moon, et al. 2012 [17]	01 With pulmonary metastasis	54 year	Male	Left	2^nd^ metacarpal	Curettage followed with chemotherapy (adriamycin and cisplatin)
11	Keith Jackson, et al. 2012 [18]	01 Recurrent after 43 years	69 year	Male	Right	4^th^ metacarpal	Intralesional excision and autogenous bone grafting
12	Lalit Maini, et al. 2011 [19]	01	25 year	Female	Right	5^th^ metacarpal	Enbloc resection of the tumor with free osteoarticular metatarsal transfer
13	Salim Al Lahham, et al. 2013 [20]	01	06 year	Female	Left	5^th^ metacarpal	Complete excision with the reconstruction of the defect with 2nd phalanx of the third toe
14	Nash H. Naam, et al. 2013 [21]	01	25 year	Female	Left	4^th^ and 5^th^ metacarpal	Wide local excision and ray amputation
15	Soobin Lim et al. 2016 [22]	01	63 year	Female	Right	1^st^ metacarpal	Stage I – excision-bone cement spacer with external fixator application; Stage II – Tricortical iliac crest graft with adjacent joint fusion
16	Athanasian EA, 2004 [23]	01	51 year	Male	Right	5^th^ metacarpal	Reconstruction of the entire fifth metacarpal bone after en-bloc resection with a Y-shaped bone fusion to the fourth metacarpal
17	Paweł Reichert, et al. 2017 [24]	01	25 year	Female	Left	1^st^ metacarpal	Stage I – excision of the tumor with external fixator application; Stage II – the corticocancellous bone graft from the iliac crest with k wire fixation
18	Thipachart Punyaratabandhu, et al. 2017 [25]	01	37 year	Female	Left	1^st^ metacarpal	Stage I – excision of the first metacarpal with cement spacer application; Stage II – titanium prosthesis application with ligament reconstruction
19	Laura W, et al. 2017 [26]	01	57 year	Female	Right	4^th^ metacarpal	Excision of the tumor with 1 cm safe margin and fresh-frozen allograft metacarpal
20	Pankaj Kumar Mishra, et al. 2017 [27]	01	13 year	Female	Right	5^th^ metacarpal	Free osteoarticular metatarsal transfer
21	Bokemper MK, et al. 2016 [28]	01	23 year	Male	Right	3^rd^ metacarpal	Third-ray resection and limited midcarpal fusion
22	Kabul C Saikia, et al. 2011 [29]	02	24 year	Female	Left	4^th^ metacarpal	Ray resection of the ring finger
			49 year	Male	Right	1^st^ metacarpal	Excision of the tumor with tricortical iliac crest graft

In 2012, Jin Chang Moon et al. reported a rare case of second metacarpal GCT in a 54-year-old patient, which was managed with chemotherapy with excellent results [17]. In the same year, Keith Jackson et al. reported one case of recurrence of fourth metacarpal GCT after 43 years of initial treatment, representing the versatility of GCT [18].

After curettage followed by autologous bone graft, around 90% of recurrence was reported [3], leading to extensive en-block excision of the tumor as the only option [4]. Procedures like local resection or ray amputation are used to eradicate the disease but long-term follow-up results are still not available in the literature.

Maini et al. tried to preserve the joint function of the fifth metacarpal giant cell tumor; they did en-bloc resection of the tumor with the fifth metacarpal and regained joint function with free osteoarticular metatarsal with the joint capsule, synovium, and ligaments transfer in a single-stage surgery. They hypothesized that the synovial layer of the proximal phalanx provides continuous nutrition to cartilage and metatarsal head, which enhances graft uptake [19].

Patrick S et al. used an autologous tricortical iliac graft after ray amputation of metacarpal giant cell tumor [14]; similarly, Manfrini et al. managed recurrent giant cell tumor by en-block resection and autologous fibula graft with implant arthroplasty at the metacarpophalangeal joint and observed excellent hand function in eight-year follow-up [9]. We reported a rare case of entire first metacarpal GCT in an around 29-year-old female, which was managed with en-block resection of the tumor with interposition of free fibular bone graft and MCP and carpometacarpal joint arthrodesis. This option allowed salvage of the patient’s native thumb with functional use as a stable post, to which she can pinch and grasp objects.

In contrast to implants, heavy plates, and screws interfering with tendon gliding, we could achieve adjacent metacarpophalangeal and carpometacarpal joint fusion with the help of only K wire and supplemented plaster support. We are reporting the case for its size (mammoth), total excision of the thumb metacarpal, fibula graft reconstruction, transarticular K wire arthrodesis, and its rarity.

## Conclusions

Giant cell tumor of the metacarpal is a rare type, with rapid progression of signs and symptoms even in young patients. They are osteolytic locally aggressive tumors with a high incidence of metastasis and recurrence. En-bloc resections of tumors with a 1 cm safe margin prevent recurrence in most cases. But the loss of hand function cannot be exigent. We proposed single-stage surgical resection of the first metacarpal and reconstruction by free fibular graft reconstruction. The short-term result is very encouraging with respect to both the function and absence of recurrence and are awaiting long-term follow-up.

## References

[1] Dahlin DC, Unni KK (1996). Dahlin's Bone Tumors: General Aspects and Data on 11087 Cases. 5th Ed. https://www.worldcat.org/title/dahlins-bone-tumors-general-aspects-and-data-on-11087-cases/oclc/638759259?referer=di&ht=edition.

[2] Averill RM, Smith RJ, Campbell CJ (1980). Giant cell tumours of the bones of the hand. J Hand Surg Am.

[3] Williams J, Hodari A, Janevski P, Siddiqui A (2010). Recurrence of giant cell tumors in the hand: a prospective study. J Hand Surg Am.

[4] Dahlin DC (1987). Giant cell bearing lesion of the bone of the hands. Hand Clin.

[5] Pai SB, Lalitha RM, Prasad K, Rao SG, Harish K (2005). Giant cell tumor of the temporal bone - a case report. BMC Ear Nose Throat Disord.

[6] Utrecht VH (1965). Giant cell tumors and aneurysmal bone cysts of spine. J Bone Joint Surg.

[7] Betsy M, Kupersmith LM, Springfield DS (2004). Metaphyseal fibrous defects. J Am Acad Orthop Surg.

[8] Chatterjee A, Dholakia DB, Vaidya SV (2004). Silastic replacement of metacarpal after resection of giant cell tumour. A case report. J Hand Surg Br.

[9] Manfrini M, Stagni C, Ceruso M, Mercuri M (2004). Fibular autograft and silicone implant arthroplasty after resection of giant cell tumor of the metacarpal—a case report with 9-year follow-up. Acta Orthop Scand.

[10] Yoshida T, Sakamoto A, Tanaka K, Matsuda S, Oda Y, Iwamoto Y (2007). Alternative surgical treatment for giant-cell reparative granuloma in the metacarpal, using phenol and ethanol adjuvant therapy. J Hand Surg Am.

[11] Kotwal PP, Nagaraj C, Gupta V (2008). Vascularised joint transfer in the management of recurrent giant cell tumour of the second metacarpal. J Hand Surg Eur.

[12] Arroud M, Afifi MA, Chbani L, Riffi AA, Bouabdallah Y (2010). Giant-cell tumor of the fourth metacarpal bone in children: case report. J Pediatr Orthop B.

[13] Shahid M, Varshney M, Maheshwari V, Mubeen A, Gaur K, Siddiqui M (2011). Giant cell tumour of first metacarpal bone. BMJ Case Rep.

[14] Jaminet P, Pfau M, Greulich M (2011). Reconstruction of the second metacarpal bone with a free vascularized scapular bone flap combined with nonvascularized free osteocartilagineous grafts from both second toes: a case report. Microsurgery.

[15] Al-Kindi H, George M, Malhotra G, Al-Muzahmi K (2011). An uncommon presentation of giant cell tumor. Oman Med J.

[16] Jones NF, Dickinson BP, Hansen SL (2012). Reconstruction of an entire metacarpal and metacarpophalangeal joint using a fibular osteocutaneous free flap and silicone arthroplasty. J Hand Surg Am.

[17] Moon JC, Kim SR, Lee YC, Chung MJ (2012). Multiple pulmonary metastases from giant cell tumor of a hand. Am J Med Sci.

[18] Jackson K, Key C, Fontaine M, Pope R (2012). Recurrence of a giant cell tumor of the hand after 42 years: case report. J Hand Surg Am.

[19] Maini L, Cheema GS, Yuvarajan P, Gautam VK (2011). Free osteoarticular metatarsal transfer for giant cell tumor of metacarpal—a surgical technique. J Hand Microsurg.

[20] Al Lahham S, Al Hetmi T, Sharkawy M (2013). Management of giant cell tumor occupying the 5th metacarpal bone in 6 years old child. Qatar Med J.

[21] Naam NH, Jones SL, Floyd J, Memisoglu EI (2014). Multicentric giant cell tumor of the fourth and fifth metacarpals with lung metastases. Hand.

[22] Lim S, Babineaux KL (2016). Reconstruction of an entire thumb metacarpal: a case report. Plast Reconstr Surg Glob Open.

[23] Bergmeister KD, Kneser U, Bickert B (2017). Functional reconstruction of the entire fifth metacarpal bone after en-bloc resection with a Y-shaped bone fusion to the fourth metacarpal: a case report. J Hand Surg Eur.

[24] Reichert P, Kowalski P, Gosk J (2017). The giant cell tumour of the proximal phalanx of the thumb treated by a 2-stage operation. Acta Orthop Traumatol Turc.

[25] Punyaratabandhu T, Lohwongwatana B, Puncreobutr C, Kosiyatrakul A, Veerapan P, Luenam S (2017). A patient-matched entire first metacarpal prosthesis in treatment of giant cell tumor of bone. Case Rep Orthop.

[26] Lewallen LW, Wagner ER, Moran SL (2017). Giant cell tumor of the metacarpal: case report. Hand.

[27] Mishra PK, Agarwal Y, Singhal P, Mishra KS (2017). Giant-cell tumor of metacarpal in the skeletally immature patient and free osteoarticular metatarsal transfer: review of literature with case report. J Orthop Case Rep.

[28] Bokemper MK, Araiza ET, Templeton KJ, Fox TJ (20181). Third-ray and capitate resection with limited midcarpal fusion for recurrent giant cell tumor: a case report. JBJS Case Connect.

[29] Saikia KC, Bhuyan SK, Ahmed F, Chanda D (2011). Giant cell tumor of the metacarpal bones. Indian J Orthop.

